# Optimizing Myanmar’s community-delivered malaria volunteer model: a qualitative study of stakeholders’ perspectives

**DOI:** 10.1186/s12936-021-03612-6

**Published:** 2021-02-08

**Authors:** Win Han Oo, Elizabeth Hoban, Lisa Gold, Kyu Kyu Than, Thazin La, Aung Thi, Freya J. I. Fowkes

**Affiliations:** 1 School of Health and Social Development, Faculty of Health, Deakin University, VIC, Australia; 2 Disease Elimination Program, Burnet Institute, VIC, Australia; 3 Department of Public Health, Myanmar Ministry of Health and Sports, Nay Pyi Taw, Myanmar; 4 Melbourne School of Population and Global Health, University of Melbourne, VIC, Australia; 5 Department of Epidemiology and Preventive Medicine, Monash University, VIC, Australia

**Keywords:** Volunteer, Community-delivered model, Malaria elimination, Primary health care, Myanmar

## Abstract

**Background:**

In parallel with the change of malaria policy from control to elimination and declines in the malaria burden in Greater Mekong Sub-region, the motivation and social role of malaria volunteers has declined. To address this public health problem, in Myanmar, the role and responsibilities of malaria volunteers have been transformed into integrated community malaria volunteers (ICMV), that includes the integration of activities for five additional diseases (dengue, lymphatic filariasis, tuberculosis, HIV/AIDS and leprosy) into their current activities. However, this transformation was not evidence-based and did not consider inputs of different stakeholders. Therefore, qualitative stakeholder consultations were performed to optimize future malaria volunteer models in Myanmar.

**Methods:**

Semi-structured interviews were conducted with key health stakeholders from the Myanmar Ministry of Health and Sports (MoHS) and malaria implementing partners to obtain their perspectives on community-delivered malaria models. A qualitative descriptive approach was used to explore the experiences of the stakeholders in policymaking and programme implementation. Interview topic guides were used during the interviews and inductive thematic data analysis was performed.

**Results:**

While ICMVs successfully provided malaria services in the community, the stakeholders considered the ICMV model as not optimal and suggested that many aspects needed to be improved including better training, supervision, support, and basic health staff’s recognition for ICMVs. Stakeholders believe that the upgraded ICMV model could contribute significantly to achieving malaria elimination and universal health care in Myanmar.

**Discussion and conclusion:**

In the context of high community demand for non-malaria treatment services from volunteers, the integrated volunteer service package must be developed carefully in order to make it effective in malaria elimination programme and to contribute in Myanmar’s pathway to universal health coverage (UHC), but without harming the community. An evidenced-based, community-delivered and preferred model, that is also accepted by the MoHS, is yet to be developed to effectively contribute to achieving malaria elimination and UHC goals in Myanmar by 2030.

## Background

Since 2000, the global malaria burden has declined significantly and several countries approached malaria elimination between 2000 and 2018 [1]. While China reported zero indigenous cases, other countries in the Greater Mekong Sub-region (GMS) have progressed towards their malaria elimination targets of 2025 for *Plasmodium falciparum* and 2030 for *Plasmodium vivax*, with malaria morbidity and mortality reduced by 76% and 95%, respectively, between 2010 and 2018 [1].

Community health workers (CHWs) (also known as malaria volunteers or village malaria workers) have contributed significantly to the recent declines in the malaria burden. Malaria volunteers have been involved in providing malaria services in rural areas where the coverage of formal health services is limited [2]. The use of community-based delivery methods is increasing, because they can be implemented with minimal training and are successful and cost-effective in resource-limited countries and in several disease control programmes [3–5]. However, along with the decline in the malaria burden, the global and regional conceptual framework for malaria has changed in recent years from control to elimination [6]. In parallel, the motivation and social role (the socially constructed role and popularity of a CHW in the context of their community) of malaria volunteers have quickly plummeted along with the decline of malaria burden in GMS, including Myanmar [7].

Recruitment and training of CHWs in Myanmar was initiated in the 1980s using multiple sources of public and non-government funds. The CHWs had been primarily trained for surveillance of epidemic outbreaks, health education, helping in sanitation and immunization activities, early referral of cases to health centres and other health activities as directed by basic health staff (BHS), the Myanmar Ministry of Health and Sports (MoHS)-appointed staff who provide curative and preventive services at primary and secondary levels in the health system [8]. Volunteers for malaria control were first used in Myanmar in 2004 by Myanmar Council of Churches when it commenced a community-delivered malaria control project focusing on early diagnosis and treatment in 160 remote villages in eight townships [9]. Most CHWs in villages were renamed “malaria volunteers” and focused on malaria control activities until late 2017. During this time in Myanmar like many areas of the GMS, although the burden of non-malarial illness remains high, the malaria burden in villages has declined significantly [10–13], Consequently, the role of malaria volunteers in the community, who only provided services for malaria, diminished as evidenced by declining malaria testing rates [14]. To restore their motivation and social role, malaria volunteers were transformed into integrated community malaria volunteers (ICMVs) in 2017–2018.

Currently, over 15,000 out of 67,285 villages in Myanmar (approximately 23%) have at least one malaria volunteer/ ICMV [15]. The ICMV model integrates activities for five additional diseases (dengue, lymphatic filariasis, tuberculosis, HIV/AIDS and leprosy, Table 1), into the current activities of malaria volunteers. However, these diseases were selected due to the landscape of the MoHS and operational feasibility, but did not formally review or take into account perspectives of stakeholders from MoHS and implementing partner (IPs) nor the community (members, leaders or ICMVs). Furthermore, recent evaluations have shown that by providing additional health service incentives, such as treatment for febrile illness, childhood pneumonia and diarrhoea, to visit volunteers malaria testing rates can be improved [14, 16, 17]. Given the changing disease burden and refocus of malaria strategy from control to elimination, as well as environmental and social changes in the malaria at-risk areas, there is an opportunity for Myanmar to reconsider the composition and implementation of their community-delivered malaria volunteer model.

**Table 1:** The diseases included in the integrated community malaria volunteer (ICMV) model and interventions ICMVs provide [65]

	Disease	Interventions
1	Malaria	Prevention and health education, community mobilization for malaria activities Helping in distribution of long-lasting insecticidal nets and dipping existing bed nets Early diagnosis, treatment and referral of malaria cases according to the National Malaria Treatment Guidelines Early warning and reporting of possible malaria outbreaks in the community to the health department Data entry, compilation and reporting of rapid diagnostic test-tested malaria cases using the prescribed formats Helping in entomological, malaria elimination and community-based research activities
2	Dengue	Assisting Vector Borne Disease Control Programme staff and basic health staff (BHS) in vector control activities Helping in referral of dengue suspected patients to the nearest health centre
3	Lymphatic filariasis	Helping BHS in mass drug administration activity for lymphatic filariasis elimination Reporting of lymphatic filariasis cases to the health department and assisting in the home-based care of lymphatic filariasis cases
4	Tuberculosis (TB)	Checking for TB signs and symptoms, and referral of suspected TB patients Contact tracing of TB patients in their communities Serving as directly observed treatment providers Following up the lost-to-follow-up TB patients (defaulter tracing) Helping TB patients in follow-up sputum examinations Assisting BHS in TB health education talks and active case detection activities
5	human immunodeficiency virus / acquired immunodeficiency syndrome (HIV/ AIDS)	Providing health education on HIV/AIDS and other sexually transmitted diseases Assisting in the mitigation of discrimination against HIV/AIDS patients Informing villagers of locations of clinics where they can get free services for HIV/AIDS and other sexually transmitted diseases Helping in referral of clients who need sexually transmitted disease treatment and HIV testing (Note: ICMVs must keep HIV/AIDS and sexually transmitted diseases information confidential.)
6	Leprosy	Providing health education in the community – communicating key leprosy messages to villagers Referral of suspected leprosy cases to health departments Referral of disabled, old and new leprosy patients who are suffering from reaction and complications of leprosy Assisting BHS and leprosy programme staff to detect new leprosy cases Assisting the leprosy programme in its public health projects

The processes of development and adoption of a context-specific, government-endorsed, and evidence-based integrated model is required to achieve malaria elimination goals. To develop an evidence-based model, a systematic review [2] and thorough community consultations [18] have already been conducted. Briefly, the community identified common health problems apart from malaria, such as the flu (fever, sneezing and coughing), diarrhoea, skin infections and tuberculosis. Incorporating preventive, and whenever possible curative, services for those diseases into the current community-delivered model was recommended. However, perspectives of stakeholders from MoHS and IPs need to be included in the model development so that the chances of policy uptake by the government to the integrated model produced will be maximized. This paper describes a qualitative study exploring the views of the MoHS and its malaria control and elimination IP stakeholders on the current community-delivered malaria models in Myanmar, their experiences in policy making and programme implementation of community-delivered models, the strategies that maintain and motivate the social role of volunteers in the community, and their preferred community-delivered malaria model.

## Methods

This qualitative study employed semi-structured interviews (key informant interviews (n = 11) and in-depth interviews (n = 8)). Stakeholder perspectives were sought from Myanmar MoHS (n = 9) and IP staff (n = 10) (Table 2) using interview guides. The MoHS stakeholders included national, state/regional and township-level Department of Public Health staff. The IP stakeholders included staff from local and international non-government organizations, United Nations agencies and philanthropy/donor agencies working in the malaria and public health sectors in Myanmar. They ranged from country/executive director to field officer level. All participants were aged more than 18 years and have experience in managing community-delivered malaria models and four stakeholders were women, which is representative of the staff proportion.

**Table 2:** Summary of semi-structured interview participants

Interviews	Myanmar ministry of health and sports stakeholders		Implementing partners stakeholders		Total
	High-level national and state/regional stakeholders^a^	Middle-level national and state/regional stakeholders^b^	High-level stakeholders^c^	Middle-level stakeholders^d^	
Key informant interview	5		6		11
In-depth interview	2	2		4	8
Total	7	2	6	4	19

^a^High-level national stakeholders from Myanmar Ministry of Health and Sports provided the perspectives of high-level government health authorities, and did so with respect to the entire country. They included Vector Borne Disease Control (VBDC) Programme managers, the National Malaria Control Programme (NMCP) assistant director, and national-level staff who had formerly engaged in the malaria programme. High-level state/regional stakeholders were targeted to gather the perspectives of state/regional level government health authorities, and their views mainly reflected the state/region that they were managing. The participants included a state/regional public health director, a VBDC state/regional officer, and a VBDC team leader

^b^Middle-level national stakeholders were targeted to learn the experiences and perspectives of middle-level government health staff. They spoke mainly about their personal experiences and assignments at the national level. The participants included a VBDC medical officer and NMCP officer. Middle-level state/regional stakeholders gave information about the experiences and perspectives of middle-level government health staff at state/regional level. They reflected their personal experiences and assigned territories. They included a district public health officer, malaria assistants at state/regional level, and middle-level VBDC focal staff at state/regional level

^c^High-level stakeholders from implementing partners contributed their perspectives about their organizations’ malaria programmes. They included a programme director, deputy programme director, programme coordinator, technical lead, and technical director

^d^Middle-level stakeholders from implementing partners provided perspectives that reflected their assigned sub-division in the programme and/or assigned geographic areas. They included a programme manager, deputy programme manager, project manager and technical supervisor

A qualitative descriptive approach was used to explore the experiences of the stakeholders in policymaking and programme implementation [19] of community-delivered models. One-on-one face-to-face interviews enabled the researcher to obtain rich qualitative information from the participants. Interviews extracted a combination of factual information (e.g., factors that need to be addressed during the control–elimination transition) and subjective information (e.g., personal experiences on managing malaria volunteers). The interviews focused on the participants’ areas of expertise, experiences in malaria programme and were tailored to the context of geographical regions where the interviewees were working.

Interview topic guides were developed and then revised following pilot testing in the field with a sample of similar participants (two key informant and two in-depth interviews) in February 2018. The participants who joined the pilot testing were not recruited again for the actual research. Topic guides were again revised during the research process by adding interview questions for emerging themes and dropping questions for saturated themes after data saturation was achieved in some themes (Additional file 1).

Given the likelihood that there would be significant variation in the perspectives of stakeholders working at different levels, the interviews were stratified and analysed into high-level and middle-level for both MoHS and IP stakeholders (Table 2). In order to collect information from a wide range of people with first-hand knowledge [20] of community-delivered models and malaria, key informant interviews were used to collect data from high-level policymakers, decision-makers and managers from Myanmar’s MoHS and malaria IPs. The in-depth interviews were conducted with middle-level managers, staff and technical advisors from Myanmar’s MoHS and malaria IPs, to take advantage of their knowledge and personal experiences in managing, implementation and operating the community-delivered models and malaria interventions [19, 21].

The participant decided on the time and location for the interview, which ensured privacy and addressed any risks associated with their participation. Interviews were conducted in participants’ workplaces and social meeting places, for example tea houses and restaurants.

The sample size for the qualitative study was based on the sample composition of diverse subgroups of stakeholders and the availability of people in each subgroup [22, 23]. Data saturation in all themes was used to determine when to cease recruitment in each subgroup [19].

From February to April 2018, the first author purposively recruited eligible participants who were provided with information that outlined the study, data collection methods (including measures to protect confidentiality), and participant’s role and responsibility in the study; this information was provided verbally and in a written information sheet (Additional file 2). Written informed consent was obtained from all participants prior to commencing the interview.

All interviews were conducted in person by the first author, other than one via telephone due to the participant’s tight schedule. Interviews were audio-recorded and field notes taken with the informed consent of the interviewees. The first author completed reflection field notes on each interview within 24 h of its completion. Interviews lasted for 45–60 min and interviewees were provided with refreshments and a small gift for their time spent in the interview.

The audio recordings were transcribed verbatim and translated into English for analysis. Inductive thematic analysis [19] was chosen because of the diverse opinions and views of the participants. The process included the steps of data immersion, coding, categorization/sub-theme development and major theme development, guided by the collected data via an in-depth code guide that was developed after data immersion and revised along with the coding process and development of themes [24]. Data collection and analysis (data immersion, coding and development of themes) were conducted in parallel and emerging themes during the data collection were captured and incorporated into the thematic framework in the data analysis stage. The level of analysis was mainly surface level, that is, exploring patterns and new understandings relating to the perspectives and experiences of the participants. The first author analysed all the data and the second author randomly extracted 10% of the data and performed an independent analysis. Afterwards, the two authors discussed the themes and subthemes and reached a consensus [25].

Key findings were illustrated with direct quotations from the data and experiences of the data collection and analysis phases were considered during the write up demonstrating reflexivity [26, 27] which improved the rigour of the study. All participants were invited to participate in member checking [28] and all undertook the task.

## Results

Four major themes were developed from this study: traditional malaria volunteer model; ICMV model; maintaining volunteers’ motivation and social role; and stakeholders’ proposed community-delivered model. There were two sub-themes to the ICMV model theme (strengths and limitations of ICMV model) and two sub-themes in the stakeholders’ proposed model theme (adaptation of current models to fit into malaria elimination programme and integration of non-malaria health services).

In the results section, the two existing models, malaria volunteer model and ICMV models, are first discussed. In the discussion of ICMV model, its strengths and limitations are included. Linking to the limitations of ICMV model, the ways to maintain volunteers’ motivation are discussed. Finally, the stakeholder proposed community-delivered model is considered.

### The traditional malaria volunteer model

The stakeholders explained that the community-delivered model for malaria services administered in Myanmar prior to transition to the ICMV model was the traditional CHW model, also known as the malaria volunteer model. Volunteers are usually selected by basic health staff (BHS) and/or IP’s field staff and trained by NMCP or IP. They are technically and logistically supported and supervised by BHS or field staff from the IP who trained them. Malaria volunteers mainly work in hard-to-reach areas that lack formal health service coverage and complement the formal health sector rather than compete with it.

With the transition of malaria control to elimination, the roles and priorities of malaria volunteers are changing, demanding higher-quality technical skills and more effort on the part of volunteers. The human resource pool in malaria-endemic villages is limited, resulting in the same volunteers working in the elimination programme who have been working in control programme.

### The integrated community malaria volunteer model

In 2016–2017, national health authorities developed the ICMV model that added services for other communicable diseases (dengue, lymphatic filariasis, tuberculosis, HIV/AIDS and leprosy) in order to maintain the social role of malaria volunteers; a malaria volunteer before transitioning into ICMV has no role if there is no malaria in the village. In 2018–2019 the ICMV model (Table 1) expanded nationally. Although implementation of the ICMV model had just commenced in some areas in Myanmar at the time of the study, the stakeholders were able to discuss the strengths and limitations of the model based on their experience during the interviews.

### Strengths of ICMV model

Stakeholders perceived that a benefit of moving to an ICMV model is that the population has access to services for many diseases that cause fever, whereas malaria volunteers (in theory) only address malaria. ICMVs are trained and equipped with tools for diagnosis of non-malaria communicable diseases (Table 1).

Good points are … if the diseases presenting with fever are combined in this model … like TB and malaria … the feverish patients go to the volunteers, then they [ICMVs] can do a blood test for malaria and can give them some advice about TB. (Middle-level MoHS stakeholder).

Although ICMVs provide interventions for other communicable diseases in the community, they are not allowed to prescribe antibiotics to patients for the treatment of infections, instead, they are required to refer non-malaria patients to BHS; this referral strategy prevents ICMVs from working as “*quacks*” (the nearest English translation of a Burmese term for an unqualified provider of medical services and medications usually provided by a doctor that often cause harm to the health of patients).

*In my opinion, I guess, they [MoHS stakeholders] have already prevented a side effect [a side effect of prescribing antibiotics by ICMVs is the risk of ICMVs transforming into quacks]. They [the ICMVs] are not allowed to use antibiotics in the ICMV model.”* (High-level IP stakeholder).

Interviewees argued that the transition of malaria volunteers into ICMVs will maintain the social role of volunteers in the community.

*“I think it is good because these diseases are actually common in the community, but they are the forgotten diseases … When they are labelled well in the ICMV model, these diseases will become noticeable. They [volunteers] exist as the frontline providers”* (Middle-level IP stakeholder).

From a programmatic perspective, the ICMV model is expected to achieve targets for the other five non-malaria disease control programmes. *“We taught them the signs and symptoms of TB. They refer suspected clients for sputum examination. They can help in TB active case-finding activities. Moreover, leprosy is in the model and so it is good for the programme”* (Middle-level MoHS stakeholder).

### Limitations and challenges of the ICMV model

The changing scope of the malaria volunteers’ role and their increased responsibilities creates limitations and challenges to the expanded ICMV model. Important limitations stated by both high and middle-level stakeholders include the increased workload for ICMVs and the difficulty of providing consistent and equitable attention to all services across the ICMV model. The current malaria control and elimination activities take up a lot of the ICMVs’ time and the middle-level interviewees were concerned that adding an additional five diseases to the ICMV’s scope of work will increase pressure on the volunteers and increase the attrition rate.

*People said the volunteers were not doing well even when their assignments were only for malaria. It will get worse if the tasks for other diseases [in the ICMV model] are appended to their current job description. I am sure they will give up. So, it will get worse.* (Middle-level MoHS stakeholder).

Some middle-level interviewees argued that the ICMVs’ contribution to malaria elimination activities will be compromised or limited because of their engagement in non-malaria programmes, noted above. *“Now, they have to share their efforts across six programmes. How can they focus on malaria?”* (Middle-level IP stakeholder). There were similar concerns from high-level stakeholders about the quality of ICMVs’ work in the non-malaria disease control programmes, stemming from an assumption that ICMVs’ performance is limited due to their low education level and the reduced time available to provide quality services across all diseases. One concern raised by high-level stakeholders was the requirement that ICMVs screen suspected cases of TB using the national screening guidelines and then refer all suspected cases to health centres for confirmation of diagnosis. If this were to occur, they considered that many patients would be misdiagnosed and have to pay unnecessary travel costs to and from health centres. Middle-level stakeholders also felt that the BHS would be overburdened with additional clients referred by ICMVs.

*We also need to think about the burden on the health care providers. I can’t comment on other areas, but in the self-administered zone, there is only one centre that can diagnose and initiate TB treatment. Only one doctor is there. If ICMVs refer suspected TB clients without proper screening, then, he [the doctor] will be angry.* (Middle-level IP stakeholder).

As noted above, both high and middle-level interviewees worried that ICMVs may work as quacks because some had worked as a quack before being recruited as a malaria volunteer: *“… the downside is that the volunteers will turn into quacks if they are not supervised closely”* (Middle-level MoHS stakeholder). *“As a potential side-effect, I would say, they might turn into quacks. I am worried about this”* (Middle-level MoHS stakeholder). The community’s expectation for treatment services from the volunteers is significant. *“Even when they [the volunteers] try to refer, people ask for treatment services”* (Middle-level MoHS stakeholder). Another interviewee said:

*We have to tell the volunteers ahead of time [in the training] that they should not work as quacks. This is the only option. It is not easy to take action and punish them if they work as quacks. Anyway, we have to take the risk and test the new model. Let’s see.* (Middle-level MoHS stakeholder).

High-level stakeholders suggested that intensive supervision and regulation by the ICMVs’ immediate supervisors, BHS and field staff, could mitigate the risk of ICMVs becoming quacks or ICMVs maintaining their quack services.

### Maintaining volunteers’ motivation and social role

The MoHS and IP stakeholders suggested ways to maintain the motivation and social role of malaria volunteers. Recommendations included educating community leaders, members, and volunteers about the concept of malaria elimination, as well as transforming the malaria volunteers into ICMVs with greater support and recognition. Nevertheless, a high-level stakeholder claims that coordinated and synchronized efforts from all IPs are required for effective health care model reform.

*If a paradigm shift is going to be made, all donors and stakeholders need to sit together beforehand and must have made allocation of responsibilities. Who will be responsible for training and who will be for supply chain, etcetera? Only with the accountability and commitments, the change should be initiated. Otherwise, the transition into ICMV model will stop after publishing the ICMV manual.* (High-level IP stakeholder).

Educating the community leaders and members about the concept of malaria elimination is essential. Community leaders and members should be informed that the majority of blood tests for malaria in the elimination phase will be negative, but that it is important to continue screening for the success of the elimination programme. They need to be taught the definition of malaria elimination, the benefits of achieving it, and the malaria elimination activities in which they can participate in simple and straightforward language. Malaria volunteers must also be educated and be advocates for the malaria elimination programme. A middle-level IP stakeholder said:

*The volunteers must receive explanations like ‘We can’t be careless. The disease burden is declining, but one positive case can spread drug-resistant malaria if we don’t diagnose and cure this case properly.’*

Both high and middle-level stakeholders perceived that educating the communities and volunteers will improve their understanding of the requirements of the malaria elimination programme and their participation in its activities. The social role of malaria volunteers will be restored because the community members will understand and appreciate the volunteers’ role in the programme. Middle-level stakeholders close to the community though that transforming the malaria volunteer model into the ICMV model will help, because ICMVs can provide multiple primary health services that the community members need. *“Now, we are going for the ICMV model … By doing so, we will be able to maintain the volunteer’s social role in the community”* (Middle-level IP stakeholder).

However, middle-level stakeholders highlighted that simply rebranding malaria volunteers as ICMVs without actually changing the service provision is not enough. In order to provide complete service package of ICMV, the volunteer support scheme that includes financial and in-kind support needs to be improved to match the increasing roles and responsibilities inherent in the ICMV model. In addition, ICMVs should be supported with resources to implement non-malaria activities in the field. To date, malaria volunteers have worked well in the malaria programme because they are provided with adequate supplies of medicines, education materials and reporting equipment. Therefore, ICMVs working for non-malaria programmes must receive adequate supplies. *“Moreover, for example, we support adequate materials for malaria, so, they [ICMVs] will need materials for TB as well. This request is logical, and we must fulfil it”* (Middle-level MoHS stakeholder).

Interviewees stated that volunteer support mechanisms need to be strengthened and the volunteer recognition system improved in the ICMV model to ensure that volunteers remain motivated. They agreed that the role, responsibilities, and contributions of volunteers in the malaria and other disease control programmes needs to be recognized by IPs and MoHS. They said that volunteers’ work can be recognized in many ways, such as awarding certificates for outstanding malaria volunteer work in their villages; provision of training completion certificates to all volunteers who attended NMCP or IP malaria training; organizing malaria refresher training in government health facilities so that the volunteers feel included in the health system; and MoHS senior officials and IP senior staff conducting joint supportive supervision visits.

### Stakeholders’ proposed community-delivered model

The MoHS and IP stakeholders’ preferences for a community-delivered model centred on two transitions: from malaria control to elimination volunteers, which incorporates additional elimination activities, and from malaria elimination to integrated elimination volunteers, in which interventions for non-malaria ICMV communicable diseases are added to the volunteer’s job description. During the analysis, the interviewees’ individual preferences were found to converge on a coherent management structure.

### Adaptation of current models to fit into malaria elimination programme

Both high and middle-level stakeholders suggested that in order to improve the community-delivered models all current malaria volunteers and those that have transitioned to ICMVs should be trained in malaria elimination concepts and their new role and be informed about the activities in the elimination programme. Malaria elimination concepts should be reinforced with the volunteers during field staff supervision visits so that volunteers fully understand and participate in the malaria elimination programme. The programmatic role of malaria volunteers will increase in the elimination phase due to the implementation of additional tasks.

Although volunteers typically report malaria case data monthly, the volunteers’ primary role in the elimination programme is real-time or near real-time notification of malaria cases (notification of malaria cases within 24 h after diagnosis). This surveillance role will be crucial to achieve effective implementation of other elimination activities, such as case investigation within 3 days of confirmation and foci investigation and responses within seven days of confirmation by BHS or IP staff as Myanmar adopts the 1-3-7 malaria elimination strategy [29–31].

*If there is no notification from volunteers, BHS can do nothing. So, it is very crucial to notify within twenty-four hours of blood testing. If the volunteer didn’t notify in time, the onward transmission will happen, and the malaria elimination can’t be achieved.* (High-level IP stakeholder).

For improved surveillance, an electronic reporting system needs to replace the current paper-based reporting system. Volunteers have been diagnosing and treating malaria successfully, and they can perform the surveillance function using mobile devices equally as well, with the appropriate training and support from the IPs and MoHS.

Interviewees agreed that malaria volunteers who need to travel from their community, which may lack internet access, to a location with mobile phone network coverage to complete their reporting requirements should be compensated for transportation costs. Furthermore, volunteers need to have their monthly phone bill top-up covered by MoHS or IP so they can use the internet and send SMS or phone calls to NMCP staff and BHS for reporting. *“For them [volunteers] …. for example, the phone bill should be supported to call us or to use the internet”* (Middle-level MoHS stakeholder). Another middle-level MoHS stakeholder said:

*The MoHS staff are equipped with mobile tablets for reporting. So, the volunteers should also be supported with tablets or smartphones for application-based reporting. We must think how they [volunteers] can report in time if there is no network coverage in their villages. We shall support the transportation cost for their reporting.*

Interviewees argued that apart from their primary case notification role, the volunteers should participate as team members in the case investigation and foci investigations. *“They [volunteers] will not be the team leader for these tasks [case investigation and foci investigation]. But they will still be the team members in the investigation team and foci investigation team”* (Middle-level MoHS stakeholder).

A volunteer, as a member of a team, will act as translator (to and from Burmese and the local dialect) to enable the case and foci investigation teams and community members to communicate. *“As usual, they can be the interpreters for the case investigation team if there is a language barrier”* (Middle-level MoHS stakeholder). It is anticipated that volunteers will serve as local guides in the community and facilitate case investigations, foci investigations and response activities, in particular for local advocacy related to elimination activities and mosquito breeding site management. *“In the investigation of mosquito breeding places, they [the volunteers] can contribute …. as they are local people and they know the situation very well”* (Middle-level MoHS stakeholder).

Finally, volunteers are expected to undertake surveillance of people movement in their areas because they need to watch people living near confirmed cases of malaria. Volunteers know the local context and people’s migration patterns well and are in the best position to monitor people’s movements for the purposes of malaria elimination. *“The roles of the volunteers are … monitoring the people who go outside and inside of the village. The main point is … case investigation is very important”* (Middle-level IP stakeholder).

### Integration of non-malaria health services into the current malaria volunteer model

High level stakeholders perceived that the current ICMV package reflects the epidemiology of significant communicable diseases in rural areas of Myanmar, complements existing health services and follows current MoHS policies. The assignment of volunteers to provide referral and prevention services for the five non-malaria diseases in the ICMV model is directly related to the capacity of the available volunteers and the community’s needs. However, the ICMV model needs to be reformed to build on its strengths and overcome or minimize its limitations, to improve its efficacy.

Although most stakeholders agreed that the current concept of an integrative approach applied in the ICMV model is optimal, some suggested different combinations of diseases should be included in the model. They argued that these diseases should be covered in the integrated community-delivered model in the elimination phase depending on the geographical location of the service. For example, an IP stakeholder working in ethnic health organization-controlled areas suggested the inclusion of both prevention and treatment services for acute respiratory tract infection and diarrhoea in the integrated community-delivered model, because these diseases are highly prevalent in rural areas and community demand for these services is high*.* These interviewees asserted that current malaria volunteers have the capacity to provide prevention and treatment services for these diseases if they are trained and supported to do so.

*For services … not like ICMV. In reality the common diseases … let’s say … such as acute respiratory tract infection and diarrhoea. I know the volunteers can give the treatment for these diseases and people in the villages need services for those diseases. I mean both prevention and treatment.* (Middle-level IP stakeholder).

## Discussion

### Summary of findings

While, the malaria volunteer model has performed well in the control phase, there are still aspects of the malaria volunteer model that need improvement in the elimination phase. To improve the implementation of the community-delivered malaria volunteer model, the ICMV model is currently being implemented in Myanmar. Qualitative findings identified issues in the ICMV model that need attention, such as the increased non-malaria workload placed on ICMVs and a reduced concentration on malaria elimination activities. Stakeholders suggested to improve ICMV implementation by educating community members and volunteers about malaria elimination concepts and transforming all malaria volunteers into integrated volunteers who will be provided with optimal training, monetary, in-kind and moral support. In principle, all stakeholders agreed that the improved ICMV model with support was the most appropriate model for malaria elimination and control of other communicable diseases.

### ICMV model for the malaria elimination programme

Stakeholders were concerned about the decreasing performance and quality of service provided by the ICMVs in the malaria elimination programme due to the additional non-malaria assignments in the volunteer service package. As the ICMVs’ main contributions in the malaria elimination programme are diagnosis and surveillance of malaria cases in the community, declining performance of ICMVs would be manifested as declining rapid diagnostic test (RDT) rate performed by ICMVs. Any declines in testing rates will impact the national malaria elimination programme target of an annual blood examination rate of at least 5%, but preferably 10% for populations at risk [31]. The surveillance function for RDT-detectable malaria cases, the treatment and prevention interventions of volunteers must be maintained in order to contribute to “Pillar 1: Ensure universal access to malaria prevention, diagnosis and treatment” and “Pillar 3: Transform malaria surveillance into a core intervention” in the Global Technical Strategy Framework for malaria elimination [32] regardless of the integration of other diseases into malaria volunteer model.

Although volunteers will conduct surveillance activities for RDT-detectable clinical malaria in the elimination programme, undetected subclinical malaria may sustain malaria transmission in the population [33–35] and could be targeted to advance malaria elimination in a region [34, 36, 37]. The interviewer probed the stakeholders during interviews to explore subclinical malaria. Although they understood the concept of onward transmission of subclinical malaria, they did not elaborate on potential issues relating to subclinical malaria nor did they suggest any interventions for its diagnosis and treatment (such as the use of highly sensitive RDT [38] for diagnosis and mass drug administration of anti-malarial drugs for elimination [39]) although the latter has been shown to be highly effective in Myanmar [40]. This may be because the National Plan for Malaria Elimination in Myanmar 2016–2030 does not recommend using molecular methods, highly sensitive RDT or mass drug administration. Instead, it only recommends that people with a history of fever in the past few weeks and clinically suspected asymptomatic infections are recommended to get tested for parasitaemia [31]. Therefore, optimal tools and strategies to detect, treat and follow up subclinical malaria cases tailored to Myanmar’s malaria elimination programme are yet to be implemented.

All the stakeholders were aware of challenges for *P. vivax* elimination compared to *P. falciparum* elimination [41, 42]. This is reflected in the National Plan for Malaria Elimination in Myanmar setting goals of *P. falciparum* elimination by 2025 and malaria elimination (all species) by 2030 [31]. Nevertheless, stakeholders did not recommend specific interventions for *P. vivax* elimination which may be due to possible introduction of new tools or regimens such as tafenoquine [43] or 7-day high-dose treatment regimen of primaquine [44] (current policy in Myanmar is 14-day regimen of primaquine) that are beyond the scope of development of the community-delivered malaria elimination model. If tafenoquine or 7-day high-dose primaquine regimen becomes policy in Myanmar, the stakeholders may consider including these regimes for radical cure of *P. vivax* malaria in the ICMV, or other, model.

### ICMV model in the context of primary health care in Myanmar

Stakeholders noticed that diagnosis, treatment, and surveillance of malaria by volunteers cannot be maintained without maintaining the community-delivered model’s popularity in the community. The changing malaria epidemiology in Myanmar has affected the service provision of a range of health care providers [45], such as general practitioners, informal health care providers and malaria volunteers [46]. The impact on malaria volunteers has been significant because general practitioners and informal providers also treat non-malaria cases in the community while malaria volunteers only treat malaria cases [8]. It would be for this reason, a consensus among stakeholders was that the scope of services of volunteers must be expanded to include basic prevention services for fever [46]. One stakeholder working in ethnic health organization-controlled areas suggested integrating prevention and treatment services for diarrhoea and acute respiratory infection in the community-delivered model. Nonetheless, there are limitations in expansion of volunteers’ services. The health services law in Myanmar, states that malaria volunteers are not allowed to provide treatment for non-malaria diseases, although they can provide prevention and referral services for other communicable and non-communicable diseases for community members; in contrast, BHS can provide treatment services for all diseases. By law, malaria volunteers are strictly prohibited from providing medical injections in order to prevent adverse events and unethical and inappropriate treatments in the community [47]. Nonetheless, some malaria volunteers may provide treatment for many diseases in their village where the rule of law is weak and the demand for treatment services in the communities is substantial as they are equated with physicians or nurses in the community, and furthermore they may have commenced these practices before starting work as a malaria volunteer [8, 48]. Volunteers are lay, minimally trained villagers who provide biomedical services unlike spiritual healers and herbal treatment providers who mainly provide non-injection and non-biomedical services. Some volunteers used to work as quacks by providing medical injections that is beyond their scope of work. Quacks have existed across Myanmar for many decades. To prevent volunteers from beginning to work as quacks, their induction training must be clear and unequivocal followed by intensive supervision and support by BHS. They need to be warned not to provide treatment services for non-malaria fever cases but to refer these cases to BHS for treatment. Nevertheless, a minimal risk of eluding volunteers from health system control is still present given the demand from community for treatment services is significant like the experiences of volunteers in other developing countries in Africa [49, 50].

Furthermore, the pace of extension of referral services from volunteers need to be in line with expansion of health facilities and BHS across rural areas in Myanmar. Being a developing country in the GMS, Myanmar’s health system, particularly human resources for health and infrastructure, is limited [51]. Stakeholders voiced concern that if only the volunteer service package is expanded with referral services without increasing the capacity of health facilities to host the referred cases, the township health system may be overburdened and collapse. Community attitude towards referral of non-malaria illnesses may turn into negative given the patients may spend out-of-pocket expenditure to visit the health centre but the available services may not be satisfactory.

In the context of high community demand for non-malaria treatment services from volunteers and having limitations in national health system, the integrated volunteer service package must be developed carefully in order to make it effective in malaria elimination programme and to contribute to Myanmar’s pathway to universal health coverage (UHC) but without harming the community. In addition to achieving malaria elimination in Myanmar at 2030, the government has targeted achieving UHC by 2030 [51]. Three key dimensions of UHC are; essential health service coverage, financial risk protection, and equity in coverage [52]. Myanmar has over 60,000 villages and currently over 15,000 are serviced by malaria volunteers/ICMVs [10]. If the current model transforms into a community-preferred and evidence-based community-delivered model, it will play a major role in the provision of basic essential health services in the rural settings of Myanmar thereby progressing UHC in Myanmar.

### The experiences and lessons-learnt in developing integrated community-delivered models in Myanmar

Nevertheless, an important cornerstone that needs to be achieved is the acceptance and endorsement of the developed evidence-based integrated model by Myanmar MoHS. In the past, in parallel to the development of the ICMV model, other community-delivered models were implemented in Myanmar and elsewhere, such as the integrated Community Case Management (iCCM) model, which proved to be effective in reducing the under-five mortality and malaria burden in many countries [16, 53–57] and the Medical Action Myanmar’s model, which proved to be effective in decreasing malaria incidence and producing an immediate and sustained increase in blood examination rates for malaria [14]. Neither the iCCM model nor Medical Action Myanmar’s model has national endorsement; instead, the Myanmar National Malaria Control Programme (NMCP) has rolled out the ICMV programme throughout Myanmar. In this study, most stakeholders continued supporting the existing ICMV model. But they also noticed that the current implementation of ICMV model was not optimal and there were many aspects to be improved. They believed that the ICMV model should continue as a popular model in the community only with greater support and recognition of volunteers by MoHS and IPs. Recognition and CHWs’ sense of belonging to the local government health department has previously been identified as the primary health system support in numerous African studies of CHW models [58–64] and these factors should be considered particularly during volunteer model reforms.

### Limitation of the study

Contextual factors, particularly time of data collection and geographical locations where stakeholders in the interviews were reflected, are important considerations in the translation and application of the study findings. The transition from the malaria volunteer to the ICMV model in Myanmar occurred in 2017 and early 2018 area by area. To transform into the ICMV model, many reforms needed to be made, such as providing training for non-malaria diseases, introduction of new recording and reporting forms, and system establishment in health facilities to accept referral of non-malaria cases in mass across Myanmar. Therefore, it took a few years to completely transform malaria volunteers into ICMVs. The data collection was in early 2018 and by that time, some volunteers have been transformed into ICMVs and some were not. As a result, the stakeholders discussed both models according to their existing knowledge of implementation. This also accounts for the different use of tense in stakeholder interviews. Different stakeholders’ opinions may be collected from stakeholders working in different states/regions of Myanmar or in another point of time of data collection as this study was conducted in 2018. Although assumption bias may have occurred triangulating qualitative data obtained from different stakeholders, this bias was mitigated by performing an inductive thematic analysis, and ensuring rigor throughout the study [19].

## Conclusion and recommendation

This qualitative study was performed in order to collect inputs from MoHS and IP stakeholders to optimize the current community-delivered malaria model in the contexts of malaria elimination programme and primary health care in Myanmar. Stakeholders believed that ICMV model needs improvement to effectively maintain the annual blood examination rate at a desired level. Nevertheless, the stakeholders didn’t discuss the specific challenges of eliminating subclinical malaria and vivax malaria in Myanmar. Furthermore, stakeholders suggested to continue implementing the ICMV model but with better support and recognition rather than performing a radical paradigm shift in Myanmar’s primary health care sector. Stakeholders also highlighted the importance of acceptance and endorsement of the developed evidence-based integrated model by Myanmar MoHS; without national endorsement and ownership, the model will not be rolled out at national scale although the model’s effectiveness is documented.

Therefore, any potential changes to the model should also incorporate both the views of stakeholders as well as the end users (ICMVs and community members) so data presented in this paper should be triangulated with the community perspectives on the same malaria elimination model [18]. Drawing on the findings from both the community and stakeholders, a community-delivered integrated malaria elimination model that is endorsed by the Myanmar MoHS should be developed, pilot tested and then scaled up nationally. Importantly, like the current ICMV model, any new model needs to be reviewed and revised periodically to reflect the changing epidemiology of diseases in rural areas and Myanmar’s dynamic political context. Beyond Myanmar, qualitative consultations on community-delivered models in other GMS countries should be considered with the view of developing country-specific community-delivered malaria elimination models.

## Supplementary Information

**Additional file 1.** Qualitative data collection tools.

**Additional file 2.** Informed consent forms.

**Additional file 3.** Approval letters from Myanmar Ministry of Health and Sports to conduct the research.

**Additional file 4.** Ethics committees certificates of approval.

## Data Availability

The datasets generated and/or analysed during the current study are not publicly available as the study collected data from specific townships and villages in Myanmar, and the information may be identifiable to particular individuals, risking a breach in confidentiality; but are available from the corresponding author on reasonable request.

## Abbreviations

BHS: Basic Health Staff
CHW: Community Health Worker
GMS: Greater Mekong Sub-region
HIV/AIDS: Human Immunodeficiency Virus / Acquired ImmunoDeficiency Syndrome
ICMV: Integrated Community Malaria Volunteer
iCCM: Integrated Community Case Management
IP: Implementing partner
MoHS: Myanmar Ministry of Health and Sports
NMCP: Myanmar National Malaria Control Programme
RDT: Rapid Diagnostic Test
TB: Tuberculosis
UHC: Universal Health Coverage
