# Supplementary material for: Optimizing Myanmar’s community-delivered malaria volunteer model: a qualitative study of stakeholders’ perspectives
Source: Malar J. 2021 Feb 8;20:79. doi: 10.1186/s12936-021-03612-6 (PMC7871594; doi:10.1186/s12936-021-03612-6)
Supplement: Supplementary file 1 — Additional file 1. Qualitative data collection tools. [file 12936_2021_3612_MOESM1_ESM.docx]

**Additional file 1: Qualitative data collection tools**

# **Qualitative research data collection tools before pretesting**

## **Interview topic guide for Key Informant Interviews (KII) and In-depth interviews (IDI)**

Semi-structured interview

- Introduction and ice breaking exercise
- Summary presentation of the study to the participants
- Non-identifiable information explained to the participants
  - Record the age, sex, organization in which she/he works, position/level in the organization without identifying the duty station, roles and responsibilities in summary

Draft themes, questions and probes

1. **Current malaria situation and control measures**
   1. Can you please tell me about the malaria situation in Myanmar?
   2. Why do you consider that the malaria trends demonstrate that there is the change in the malaria prevalence in Myanmar?
   3. What’s the situation of malaria in your assigned territory?
   4. Why do you think that the burden of malaria in your area is going up/down or plateauing?
   5. What are the current malaria control interventions and models in your area?
   6. What are the relative contributions of each model into the success of malaria control? How do you rate them and why?
   7. Who are the key stakeholders in successful malaria control in Myanmar and in your area?
2. **Views and perspectives on the current malaria CHW model**
   1. How do you think that the CHWs contribute to malaria control in Myanmar?
   2. Probe: Importance of their role in malaria control in Myanmar?
3. **Policy and strategic barriers and enablers for Myanmar malaria elimination (to omit this theme in IDI)**
   1. What are the policies, strategies or regulations that have led to the malaria elimination approach in Myanmar? How do these policies, strategies and/or strategies play a role in the malaria elimination?
   2. What policies, strategies and regulations hinder the malaria elimination in Myanmar? How do these policies, strategies and regulations cause hindrances in the malaria elimination?
4. **Operational barriers and enablers for Myanmar malaria control and elimination using community delivered models**
   1. Which approaches worked and which didn’t in applying community delivered models in the field? Why did this occur?
   2. What are the operational barriers and enablers in managing the community volunteers and the model?
   3. How do the barriers and enablers impact in the success or failure of the malaria control and elimination model? Why?
5. **Strategies to maintain the motivation and social role of CHWs in the community**
   1. How do you think the motivation and social role of CHWs in the community affects the malaria control and elimination in Myanmar?
   2. How do we maintain CHWs’ motivation and social role in the community?
   3. What are the rationales for your suggestions?
6. **The factors need to be addressed during the transition from malaria control to elimination using community delivered models**
   1. In the future, what role do you think the village health volunteers can play in the malaria elimination model? Why?
   2. How can we adapt the existing role of village health volunteers in malaria control to fit into the proposed elimination model?
   3. Which aspect of current malaria control model do you think can be strengthened in the future? In order to do so, what resources are needed?
7. **Additional topic identified by the interviewee**
   1. Is there anything you like to add or discuss? Please discuss more.

End of the interview

# **Qualitative research data collection tools after pretesting**

*(Compared to ‘before pretesting’ version, the changes in ‘after pretesting’ version are highlighted with underlines. Interview topic guide was separated into KII and IDI guides after pretesting)*

## **Interview topic guide for Key Informant Interviews (KII)**

Semi-structured interview

- Introduction and ice breaking exercise
- Summary presentation of the study and Myanmar malaria elimination package (if required) to the participants
- Non-identifiable information explained to the participants
  - Record the age, sex, organization in which she/he works (MoHS or IP organization), service years in malaria control sector, roles and responsibilities in summary

Thèmes, questions and probes

1. **Current malaria situation and control measures**
   1. Can you please tell me about the malaria situation in Myanmar?
   2. Why do you consider that the malaria trends demonstrate that there is the change in the malaria prevalence in Myanmar?
   3. What’s the situation of malaria in your assigned territory?
   4. Why do you think that the burden of malaria in your area is going up/down or plateauing?
   5. What are the current malaria control interventions and models in your area?
   6. What are the relative contributions of each model into the success of malaria control? How do you rate them and why?
   7. Who are the key stakeholders in successful malaria control in Myanmar and in your area?
2. **Views and perspectives on the current malaria CHW model**
   1. How do you think that the CHWs contribute to malaria control in Myanmar?
   2. Probe: Importance of their role in malaria control in Myanmar?
3. **Policy and strategic barriers and enablers for Myanmar malaria elimination**
   1. What are the health and non-health policies and/or strategies that have led to the malaria elimination approach in Myanmar?
   2. What are the health and non-health regulations that have led to the malaria elimination in Myanmar?
   3. How do these policies and/or strategies play a role in the malaria elimination? (Interviewer to give examples for the participant’s visualization)
   4. How do these regulations play a role in the malaria elimination?

(Interviewer to give examples for the participant’s visualization)

- 1. What policies and/or strategies (health and non-health) hinder the malaria elimination in Myanmar?
  2. What regulations (health and non-health) hinder the malaria elimination in Myanmar?
  3. How do these policies and/or strategies cause hindrances in the malaria elimination? (Interviewer to give examples for the participant’s visualization).
  4. How do these regulations cause hindrances in the malaria elimination?

(Interviewer to give examples for the participant’s visualization)

1. **Operational barriers and enablers for Myanmar malaria control and elimination using community delivered models**
   1. Which approaches worked and which didn’t in applying community delivered models in the field? Why did this occur?
   2. What are the operational barriers and enablers in managing the community volunteers and the model?
   3. How do the barriers and enablers impact in the success or failure of the malaria control and elimination model? Why?
2. **Strategies to maintain the motivation and social role of CHWs in the community**
   1. How do you think the motivation and social role of CHWs in the community affects the malaria control and elimination in Myanmar?
   2. How do we maintain CHWs’ motivation and social role in the community?
   3. How do we adapt the current CHW model and what are the side-effects and side-benefits of the adaptation?
   4. What are the rationales for your suggestions?
3. **The factors need to be addressed during the transition from malaria control to elimination using community delivered models**
   1. In the future, what role do you think the village health volunteers can play in the malaria elimination model? Why?
   2. How can we adapt the existing role of village health volunteers in malaria control to fit into the proposed elimination model?
   3. Which aspect of current malaria control model do you think can be strengthened in the future? In order to do so, what resources are needed?
4. **Malaria elimination in Myanmar**
   1. What do you like to recommend / suggest for the successful malaria elimination in Myanmar?
   2. How will you manage the CHW in your area in the future? Can you please draw an organogram for the management structure of CHW in the elimination context?
5. **Additional topic identified by the interviewee**
   1. Is there anything you like to add or discuss? Please discuss more.

End of the KII interview

## **Interview topic guide for In-depth interviews (IDI)**

Semi-structured interview

1. Introduction and ice breaking exercise
2. Summary presentation of the study and Myanmar malaria elimination package (if required) to the participants
3. Non-identifiable information explained to the participants
   - Record the age, sex, organization in which she/he works (MoHS or IP organization), service years in malaria control sector, roles and responsibilities in summary

Thèmes, questions and probes

1. **Current malaria situation and control measures**
   1. Can you please tell me about the malaria situation in Myanmar?
   2. Why do you consider that the malaria trends demonstrate that there is the change in the malaria prevalence in Myanmar?
   3. What’s the situation of malaria in your assigned territory?
   4. Why do you think that the burden of malaria in your area is going up/down or plateauing?
   5. What are the current malaria control interventions and models in your area?
   6. What are the relative contributions of each model into the success of malaria control? How do you rate them and why?
2. **Views and perspectives on the current malaria CHW model**
   1. How do you think that the CHWs contribute to malaria control in Myanmar?
   2. Probe: Importance of their role in malaria control in Myanmar?
3. **Operational barriers and enablers for Myanmar malaria control and elimination using community delivered models**
   1. Which approaches worked and which didn’t in applying community delivered models in the field? Why did this occur?
   2. What are the operational barriers and enablers in managing the community volunteers and the model?
   3. How do the barriers and enablers impact in the success or failure of the malaria control and elimination model? Why?
4. **Strategies to maintain the motivation and social role of CHWs in the community**
   1. How do you think the motivation and social role of CHWs in the community affects the malaria control and elimination in Myanmar?
   2. How do we maintain CHWs’ motivation and social role in the community?
   3. How do we adapt the current CHW model and what are the side-effects and side-benefits of the adaptation?
   4. What are the rationales for your suggestions?
5. **The factors need to be addressed during the transition from malaria control to elimination using community delivered models**
   1. In the future, what role do you think the village health volunteer s can play in the malaria elimination model? Why?
   2. How can we adapt the existing role of village health volunteers in malaria control to fit into the proposed elimination model?
   3. Which aspect of current malaria control model do you think can be strengthened in the future? In order to do so, what resources are needed?
6. **Malaria elimination in Myanmar**
   1. What do you like to recommend / suggest for the successful malaria elimination in Myanmar?
   2. How will you manage the CHW in your area in the future? Can you please draw an organogram for the management structure of CHW in the elimination context?
7. **Additional topic identified by the interviewee**
   1. Is there anything you like to add or discuss? Please discuss more.

End of the IDI interview

## **Revised interview topic guide for In-depth Interview (IDI) after saturation of some themes**

Semi-structured interview

- Introduction and ice breaking
- Summary presentation of the study to the participants
- Non-identifiable information of the participants
  - Record the age, organization in which she/he works (MoHS or IP organization), service years in malaria control sector, roles and responsibilities in summary

Thèmes, questions and probes

1. **Current malaria control/elimination interventions and models**
   1. What are the current malaria control/elimination interventions and models in your area?
   2. What are the relative contributions of each model into the success of malaria control? How do you rate them and why?
   3. Can you tell the importance of CHW role in malaria control in Myanmar?
2. **Operational barriers and enablers for Myanmar malaria control and elimination using community delivered models**
   1. Which approaches worked and which didn’t in applying community delivered models in the field? Why did this occur? How can we overcome the issues?
   2. What are the operational barriers and enablers in managing the community volunteers and the model?
   3. How do the barriers and enablers impact in the success or failure of the malaria control and elimination model? Why?
3. **Strategies to maintain the motivation and social role of CHWs in the community**
   1. How do you think the motivation and social role of CHWs in the community affects the malaria control and elimination in Myanmar?
   2. How do we maintain CHWs’ motivation and social role in the community?
   3. How do we adapt the current CHW model and what are the side-effects and side-benefits of the adaptation?
   4. What are the rationales for your suggestions?
4. **Future community delivered models for malaria elimination**
   1. In the future, what role do you think the VHVs can play in the malaria elimination model? Why?
   2. How can we adapt the existing role of VHVs in malaria control to fit into the proposed elimination model?
   3. Which aspect of current malaria control model do you think can be strengthened in the future? In order to do so, what resources are needed?
   4. How will you manage the CHW in your area in the future? Can you please draw an organogram for the management structure of CHW in the elimination context?
5. **Policy and strategic barriers and enablers for Myanmar malaria elimination**
   1. What are the health and non-health policies and/or strategies of the government and/or donors related to the malaria elimination in Myanmar?
6. **General comments for Myanmar malaria elimination**
   1. What do you like to recommend / suggest for the successful malaria elimination in Myanmar?
7. **Additional topic identified by the interviewee**
   1. Is there anything you like to add or discuss or ask? Please discuss more.

End of the IDI interview
