# Supplementary material for: Optimizing Myanmar’s community-delivered malaria volunteer model: a qualitative study of stakeholders’ perspectives
Source: Malar J. 2021 Feb 8;20:79. doi: 10.1186/s12936-021-03612-6 (PMC7871594; doi:10.1186/s12936-021-03612-6)
Supplement: Supplementary file 2 — Additional file 2. Informed consent forms. [file 12936_2021_3612_MOESM2_ESM.docx]

**Additional file 2: Informed consent forms**

**Ethics Review Committee**

**Department of Medical Research**

**Ministry of Health and Sports**

**Republic of the Union of Myanmar**

**Informed Consent Form for “Perspectives of community delivered models for the malaria elimination in Myanmar: A qualitative study”, Semi-structured interview with Ministry of Health and Sports (MoHS) stakeholders**

This informed consent form is for **MoHS stakeholders**, invited to participate in the **“Perspectives of community delivered models for the malaria elimination in Myanmar: A qualitative study”**

**Name of Principal Investigator**: Dr. Win Han Oo

**Name of Organization**: Deakin University and Burnet Institute

**Name of sponsor**: Deakin University and Burnet Institute

Title of study: “Perspectives of community delivered models for the malaria elimination in Myanmar: A qualitative study”

**PART 1: Information Sheet**

**Introduction**

I am ______________ and I work for the Burnet Institute funded project in Myanmar. I am conducting a study on “Perspectives of community delivered models for the malaria elimination in Myanmar: A qualitative study”. This consent form will provide you with some information about the study. It may contain words that you do not understand. Please ask me to explain any words or information that you do not clearly understand as we go through the form. I will also give you a signed copy (or unsigned, if you wish) to keep for your record.

**Purpose of the research**

This study is being conducted by Dr Win Han Oo for his PhD degree at Deakin University Australia, and is funded by Deakin University and Burnet Institute. Myanmar has the highest burden of malaria in the Greater Mekong Sub-region and aims to eliminate malaria by 2030. The PhD project aims to develop a community-delivered malaria elimination model that is acceptable, operational, pragmatic, evidence based and effective compared to the traditional model for malaria elimination in Myanmar.

This study aims to explore the perspectives, views and inputs of key health stakeholders and community members in Myanmar on community-delivered models for the development of community- delivered malaria elimination model. We are doing research to:

• explore the views and perspectives on the current malaria CHW models

• explore the strategies to maintain the motivation and social role of CHWs in the community

• explore the factors that need to be addressed during the transition from malaria control to elimination setting focusing on the community delivered models

We are collecting information from health staff, malaria implementing partner staff, community leaders and community members.

**Type of research intervention**

You are invited to participate in a semi-structured interview as part of this study. In this interview, we will ask questions about your working experience and opinions.

**Participant selection**

You are being invited to take part in this research because we feel that your experience and knowledge as a MoHS stakeholder can contribute much to our understanding and knowledge of community delivered models for malaria elimination in Myanmar.

**Voluntary Participation**

Your participation is voluntary. It is your right to decide whether or not you want to join the study or to stop participating at any time. You are not required to answer any questions that you are not comfortable with. Whether you choose to participate in this study or not, and any information you choose to provide to us, will not affect our relationship in any way.

**Procedure**

The interview will be conducted by one interviewer and one note-taker. The discussion will be held in a place where other people cannot hear the discussion. During this interview we will talk about the current malaria situation and control measures in your working area, views and perspectives on the current malaria Community Health Worker (CHW) models, policy and strategic barriers and enablers for Myanmar malaria elimination, operational barriers and enablers for Myanmar malaria elimination using community delivered models, strategies to maintain the motivation and social role of CHWs in the community, and factors that need to be addressed during the transition from malaria control to elimination using community delivered models. We will be taking notes and an audio recording of this interview. Your name will not be recorded in either the written notes or the audio recording.

**Duration**

The interview will take between 45 minutes and one hour.

**Confidentiality**

Information obtained in this interview will be kept confidential and will not be shared with anyone outside the study team. Your name or other identifying information will not appear in the notes from this discussion or in the final report, and only staff participating in the study will have access to the information you provide. Once you agree to participate in the study, we will ask you to choose a pseudonym (a fake name) in order to try to protect your privacy throughout your participation.

All consent forms, audio recordings, and notes from this study will be stored in a locked filing cabinet, and only study staff will have access to them. Representatives of “The Alfred Office of Ethics & Research Governance” and “Department of Medical Research Ethics Review Committee” will be able to access these documents. However, they would only do this to ensure that your privacy is being maintained and protected. We will use the audio recordings to produce a written record of our conversation. The audio recordings will then be destroyed after they have been copied to a password protected computer. The written record and any notes from this discussion will be stored securely for a period of seven years and then destroyed.

**Risk and discomforts**

During the course of the interview, you will be asked to talk about malaria, malaria control and elimination in Myanmar and community delivered models for malaria elimination. There is a small risk that you may feel uncomfortable answering these questions. However, we do not wish this to happen, and you may refuse to answer any question or not take part in the study if you feel uncomfortable answering any question(s).

**Benefits**

You will not get any direct benefit from participating in this study; however, your participation in this study will help the principal investigator Dr Win Han Oo create the community-delivered malaria elimination model that is acceptable, operational, pragmatic, evidence based and effective compared to the traditional model for malaria elimination in Myanmar.

**Incentives**

We will provide refreshment, 4000 Kyat to compensate you for your time for participating in the study and travel and related expenses if you had to travel from your residence or work station.

**Sharing the result**

Results from this study are expected to be shared nationally and internationally; however, no identifying information will be included with any of the results disseminated. You can request results of the study by contacting the Principal Investigator of the study after April 2018.

**Who to contact**

If you agree to participate in the study, you can contact the person listed below at any time if you have any questions:

Dr Win Han Oo

PhD Candidate

School of Health and Social Development

Faculty of Health, Deakin University

226,4th Floor, U Wisara Road,Wizaya Plaza, Bahan Township 11201, Yangon.

Email: owinhan@deakin.edu.au

Ph: +95-1-375785, 375763, 375727, 512693 Ext 106

For questions regarding study participants’ rights, please contact:

Secretary of the Ethics Review Committee

Department of Medical Research

No. 5 Ziwaka Road, Dagon PO Yangon, Myanmar

Phone: 01 375447- ext: 118 during office hours

For complaints please contact:

Complaints Officer

Office of Ethics & Research Governance, Alfred Health

Phone: +61 3 9076 3619, Email: [research@alfred.org.au](mailto:research@alfred.org.au)

Note: You will need to quote the following Alfred Health project number: 445/17

**PART 2: Certificate of Consent**

I have been invited to participate in research about **“Perspectives of community delivered models for the malaria elimination in Myanmar: A qualitative study”**. I understand that it will involve an interview that will take between 45 minutes and one hour. I am aware that there may be no benefit to me personally. I am given the contact details of the principal investigator. I have been informed about the rights of the participant.

Written consent:

I have read the information in this consent form. All my questions about the study and my participation in it have been answered. I understand what my involvement in the study means, and I voluntarily agree to participate, and understand that I have the right to withdraw from the study at any time without any consequences.

**Name of Participant**: _______________________

**Signature of Participant**: _______________________

**Date:** _______________________

(Day/month/year)

I have accurately read or witnessed the accurate reading of the consent form to the potential

participant, and the individual has had the opportunity to ask questions. I confirm that the individual has given consent freely.

**Name of Researcher:** _______________________

**Signature of Researcher:** _______________________

**Date:** _______________________ (Day/month/year)

**A copy of this Informed Consent Form has been provided to participant _____ (initialed by the researcher/assistant)**

**Ethics Review Committee**

**Department of Medical Research**

**Ministry of Health and Sports**

**Republic of the Union of Myanmar**

**Informed Consent Form for “Perspectives of community delivered models for the malaria elimination in Myanmar: A qualitative study”, Semi-structured interview with malaria implementing partner stakeholders**

This informed consent form is for **malaria implementing partner (IP) stakeholders**, invited to participate in the **“Perspectives of community delivered models for the malaria elimination in Myanmar: A qualitative study”**

**Name of Principal Investigator:** Dr Win Han Oo

**Name of Organization:** Deakin University and Burnet Institute

**Name of sponsor:** Deakin University and Burnet Institute

**Title of study:** “Perspectives of community delivered models for the malaria elimination in Myanmar: A qualitative study”

**PART 1: Information Sheet**

**Introduction**

I am ______________ and I work for the Burnet Institute funded project in Myanmar. I am conducting a study on “Perspectives of community delivered models for the malaria elimination in Myanmar: A qualitative study”. This consent form will provide you with some information about the study. It may contain words that you do not understand. Please ask me to explain any words or information that you do not clearly understand as we go through the form. I will also give you a signed copy (or unsigned, if you wish) to keep for your record.

**Purpose of the research**

This study is being conducted by Dr Win Han Oo for his PhD degree at Deakin University Australia, and is funded by Deakin University and Burnet Institute. Myanmar has the highest burden of malaria in the Greater Mekong Sub-region and aims to eliminate malaria by 2030. The PhD project aims to develop a community-delivered malaria elimination model that is acceptable, operational, pragmatic, evidence based and effective compared to the traditional model for malaria elimination in Myanmar.

This study aims to explore the perspectives, views and inputs of key health stakeholders and community members in Myanmar on community-delivered models for the development of community- delivered malaria elimination model. We are doing research to:

• explore the views and perspectives on the current malaria CHW models

• explore the strategies to maintain the motivation and social role of CHWs in the community

• explore the factors that need to be addressed during the transition from malaria control to elimination setting focusing on the community delivered models

We are collecting information from health staff, malaria implementing partner staff, community leaders and community members.

**Type of research intervention**

You are invited to participate in a semi-structured interview as part of this study. In this interview, we will ask questions about your working experience and opinions.

**Participant selection**

You are being invited to take part in this research because we feel that your experience and knowledge as an IP stakeholder can contribute much to our understanding and knowledge of community delivered models for malaria elimination in Myanmar.

**Voluntary Participation**

Your participation is voluntary. It is your right to decide whether or not you want to join the study or to stop participating at any time. You are not required to answer any questions that you are not comfortable with. Whether you choose to participate in this study or not, and any information you choose to provide to us, will not affect our relationship in any way.

**Procedure**

The interview will be conducted by one interviewer and one note-taker. The discussion will be held in a place where other people cannot hear the discussion. During this interview we will talk about the current malaria situation and control measures in your working area, views and perspectives on the current malaria Community Health Worker (CHW) models, policy and strategic barriers and enablers for Myanmar malaria elimination, operational barriers and enablers for Myanmar malaria elimination using community delivered models, strategies to maintain the motivation and social role of CHWs in the community, and factors that need to be addressed during the transition from malaria control to elimination using community delivered models. We will be taking notes and an audio recording of this interview. Your name will not be recorded in either the written notes or the audio recording.

**Duration**

The interview will take between 45 minutes and one hour.

**Confidentiality**

Information obtained in this interview will be kept confidential and will not be shared with anyone outside the study team. Your name or other identifying information will not appear in the notes from this discussion or in the final report, and only staff participating in the study will have access to the information you provide. Once you agree to participate in the study, we will ask you to choose a pseudonym (a fake name) in order to try to protect your privacy throughout your participation.

All consent forms, audio recordings, and notes from this study will be stored in a locked filing cabinet, and only study staff will have access to them. Representatives of “The Alfred Office of Ethics & Research Governance” and “Department of Medical Research Ethics Review Committee” will be able to access these documents. However they would only do this to ensure that your privacy is being maintained and protected. We will use the audio recordings to produce a written record of our conversation. The audio recordings will then be destroyed after they have been copied to a password protected computer. The written record and any notes from this discussion will be stored securely for a period of seven years and then destroyed.

**Risk and discomforts**

During the course of the interview, you will be asked to talk about malaria, malaria control and elimination in Myanmar and community delivered models for malaria elimination.. There is a small risk that you may feel uncomfortable answering these questions. However, we do not wish this to happen, and you may refuse to answer any question or not take part in the study if you feel uncomfortable answering any question(s).

**Benefits**

You will not get any direct benefit from participating in this study; however, your participation in this study will help the principal investigator Dr Win Han Oo create the community-delivered malaria elimination model that is acceptable, operational, pragmatic, evidence based and effective compared to the traditional model for malaria elimination in Myanmar.

**Incentives**

We will provide refreshment, 4000 Kyat to compensate you for your time for participating in the study and travel and related expenses if you had to travel from your residence or work station.

**Sharing the result**

Results from this study are expected to be shared nationally and internationally; however, no identifying information will be included with any of the results disseminated. You can request results of the study by contacting the Principal Investigator of the study after April 2018.

**Who to contact**

If you agree to participate in the study, you can contact the person listed below at any time if you have any questions:

Dr Win Han Oo

PhD Candidate

School of Health and Social Development

Faculty of Health, Deakin University

226,4th Floor, U Wisara Road,Wizaya Plaza, Bahan Township 11201, Yangon.

Email: owinhan@deakin.edu.au

Ph: +95-1-375785, 375763, 375727, 512693 Ext 106

For questions regarding study participants’ rights, please contact:

Secretary of the Ethics Review Committee

Department of Medical Research

No. 5 Ziwaka Road, Dagon PO Yangon, Myanmar

Phone: 01 375447- ext: 118 during office hours

For complaints please contact:

Complaints Officer

Office of Ethics & Research Governance, Alfred Health

Phone: +61 3 9076 3619, Email: [research@alfred.org.au](mailto:research@alfred.org.au)

Note: You will need to quote the following Alfred Health project number: 445/17

**PART 2: Certificate of Consent**

I have been invited to participate in research about **“Perspectives of community delivered models for the malaria elimination in Myanmar: A qualitative study”**. I understand that it will involve an interview that will take between 45 minutes and one hour. I am aware that there may be no benefit to me personally. I am given the contact details of the principal investigator. I have been informed about the rights of the participant.

Written consent:

I have read the information in this consent form. All my questions about the study and my participation in it have been answered. I understand what my involvement in the study means, and I voluntarily agree to participate, and understand that I have the right to withdraw from the study at any time without any consequences.

**Name of Participant:** _______________________

**Signature of Participant:** _______________________

**Date:** _______________________

(Day/month/year)

I have accurately read or witnessed the accurate reading of the consent form to the potential

participant, and the individual has had the opportunity to ask questions. I confirm that the individual has given consent freely.

**Name of Researcher:** _______________________

**Signature of Researcher:** _______________________

**Date:**  _______________________ (Day/month/year)

**A copy of this Informed Consent Form has been provided to participant _____ (initialed by the researcher/assistant)**
